# The ecological niche and population history shape mosquito population genetics on a group of three Caribbean islands

**DOI:** 10.1186/s13071-025-06801-3

**Published:** 2025-05-09

**Authors:** Pepijn Helleman, Maarten Schrama, Krijn B. Trimbos, Marieta A. H. Braks, Francis Schaffner, Arjan Stroo, Roel M. Wouters, Jordy G. van der Beek

**Affiliations:** 1 Institute of Environmental Sciences, Leiden University, 2333 CC Leiden, The Netherlands; 2 Biodiversity and Society Research Group, Naturalis Biodiversity Center, 2333 CR Leiden, The Netherlands; 3 Centre for Infectious Disease Control, National Institute for Public Health and the Environment (RIVM), 3721 MA Bilthoven, The Netherlands; 4 Francis Schaffner Consultancy, 4125 Riehen, Switzerland; 5 Centre for Monitoring of Vectors (CMV), Netherlands Institute for Vectors Invasive Plants and Plant Health (NIVIP), Netherlands Food and Consumer Product Safety Authority (NVWA), 6706 EA Wageningen, The Netherlands; 6 Department of Ecology, Faculty of Science, Charles University, 12844 Prague, Czechia; 7 Pandemic and Disaster Preparedness Center, Delft, Rotterdam, The Netherlands

**Keywords:** Mosquitoes, Genetic diversity, Population structure, Haplotype network, Dispersal, Mitochondrial DNA, Introduced species, Dutch Caribbean

## Abstract

**Background:**

While studies on mosquito population genetics have primarily focused on medically relevant species, fewer have examined the genetic population structure of mosquitoes from a diverse range of species within a single geographical area. The limited comparison between native and non-native species, as well as ecologically divergent species from the same region, hampers our ability to generalise previously described patterns in mosquito population genetics. This study uses the mosquito fauna of the Caribbean islands of Aruba, Curaçao and Bonaire as a case study to explore population genetic variation among both native and non-native mosquito species, as well as among native species occupying different ecological niches. We examine how genetic patterns relate to their population history and species-specific ecologies.

**Methods:**

Mitochondrial *COII* sequences were obtained from 258 mosquito specimens belonging to six species, occurring on all three islands. Sequences were used in haplotype network analysis to assess the genetic variation between mosquito populations of each of the six ecologically diverse species, which vary in both their population history and ecological niche.

**Results:**

Both the genetic diversity and population genetic structure were found to differ strongly between sets of species, leading to a subdivision into three species groups: (1) non-native species with low genetic diversity across all three investigated islands, (2) locally native species with high genetic diversity and closely related haplotypes occurring on different islands and (3) locally native species with high genetic diversity and locally restricted haplotypes.

**Conclusions:**

Our results show that the population genetics of non-native and native species strongly differ, likely as a result of population history. Furthermore, the results suggest that mosquito species sharing the same area may display distinct population genetic structure, likely related to differences in their ecology and dispersal capacity. We suggest that addressing a broader range of species within a single area will benefit future research on mosquito population genetics to place observed patterns into a broader historical, ecological and evolutionary context.

**Graphical Abstract:**

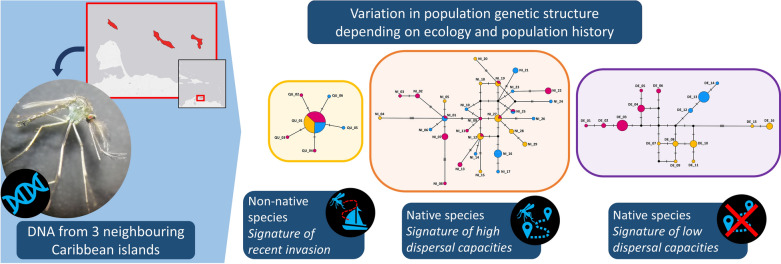

**Supplementary Information:**

The online version contains supplementary material available at 10.1186/s13071-025-06801-3.

## Background

Globally, there are approximately 3700 species of mosquitoes (Diptera: Culicidae) [1]. Although much is known about the ecology and distribution of a limited number of species, most notably the species of medical importance (e.g., [2–6]), the majority of species is greatly understudied. Similarly, mosquito population dynamics and structure have been studied using population genetics almost exclusively in relation to pathogen transmission and vector control (e.g., [7–10]). As a result of the strong focus on major invasive or medically relevant species, most information on mosquito population genetics is based on a small subset of mosquito biodiversity, representing only a small fraction of the ecological diversity among mosquitoes at a given location. Comparative population genetic studies that examine mosquitoes from different ecological backgrounds within a single location (e.g., [10, 11]) are less common compared with studies focusing on a single species or species complex (e.g., [7–9, 12–23]). Hence, it remains relatively unclear how patterns of population genetics of mosquitoes differ in structure and complexity among species with different ecological strategies that co-occur within a landscape.

Besides ecological differences, the history of a population at a specific location also leaves a genetic fingerprint. Emerging evidence demonstrates that commonly introduced species, such as *Culex quinquefasciatus* Say, 1823 and *Aedes aegypti* (Linnaeus, 1762), exhibit noticeable differences in both ecology and population genetic structure compared with native species. Recent studies often find a reduction in genetic diversity among introduced mosquito populations outside their native range compared with their source populations, which display a much higher degree of genetic diversity [11, 24–30]. By contrast, among the few studies on locally native species there are notable observations of local genetic differentiation and speciation (e.g., in the *Anopheles punctulatus* group on the Solomon Islands [31], in the *Ae. taeniorhynchus* (Wiedemann, 1821) population on the Galápagos Islands [32] and in *Cx. fuscanus* Wiedemann, 1820 populations in India, [33]). These observations of local genetic differentiation indicate a potential contrast between the non-native and the locally native mosquito species, suggesting profoundly different patterns in population genetic structure among these groups. Studying the population genetics of a variety of mosquito species in a single area allows for direct comparison of the population genetic structure of different species in relation to their behaviour and ecology, since the species studied will have been subjected to similar macro-environmental conditions. Hence, to elucidate how natural variation in population genetics relates to mosquito species ecology, patterns in genetic structure need to be studied across a more diverse assemblage of mosquito species in a single area.

For this purpose, islands of moderate size offer compelling model systems due to several advantages. Such islands provide the opportunity to sample populations across their entire local distribution, presenting a more complete and reliable representation of the overall population structure. Moreover, the surrounding ocean likely isolates the islands from most natural colonisation events by mosquitoes [34, 35]. The Dutch Leeward Antilles, comprising Aruba, Curaçao and Bonaire, provide an ideal study case to investigate the variation in mosquito population genetics, due to the presence of a patchy mosaic of various distinct habitat types on these islands (e.g., [36]) in combination with a rich local mosquito fauna consisting of both native and non-native species [37].

The objective of this study is to explore the variation in mosquito population genetics by comparing the genetic diversity and population genetic structure among a comprehensive assemblage of native and non-native mosquito species, representing 60% of the species found on all three islands (Additional File 1: Supplementary Table 2). By analysing both native and non-native mosquito species with a broad range of ecological niches, we aim to gain new insights on the role of species-specific ecology in mosquito population genetics. We hypothesise that, in a given area, population genetics of mosquitoes differ along both a historical and an ecological axis. More specifically, we expect (1) that non-native species (i.e., having a relatively short population history on the island) exhibit a much smaller genetic differentiation, resulting from less time to accumulate new mutations, a strong bottleneck effect during the introduction, and their high connectivity through easy human-mediated dispersal, and (2) that native species with a stricter ecological niche comprise more unique haplotypes compared with generalists, because their populations are more easily fragmented when suitable habitat has a patchy spatial distribution. To achieve the objectives, we used mitochondrial *COII* (or *COX2*, cytochrome c oxidase subunit 2) sequences to perform haplotype network analysis for six diverse species of mosquitoes in the southern Caribbean.

## Methods

### Study site

**Fig. 1:**
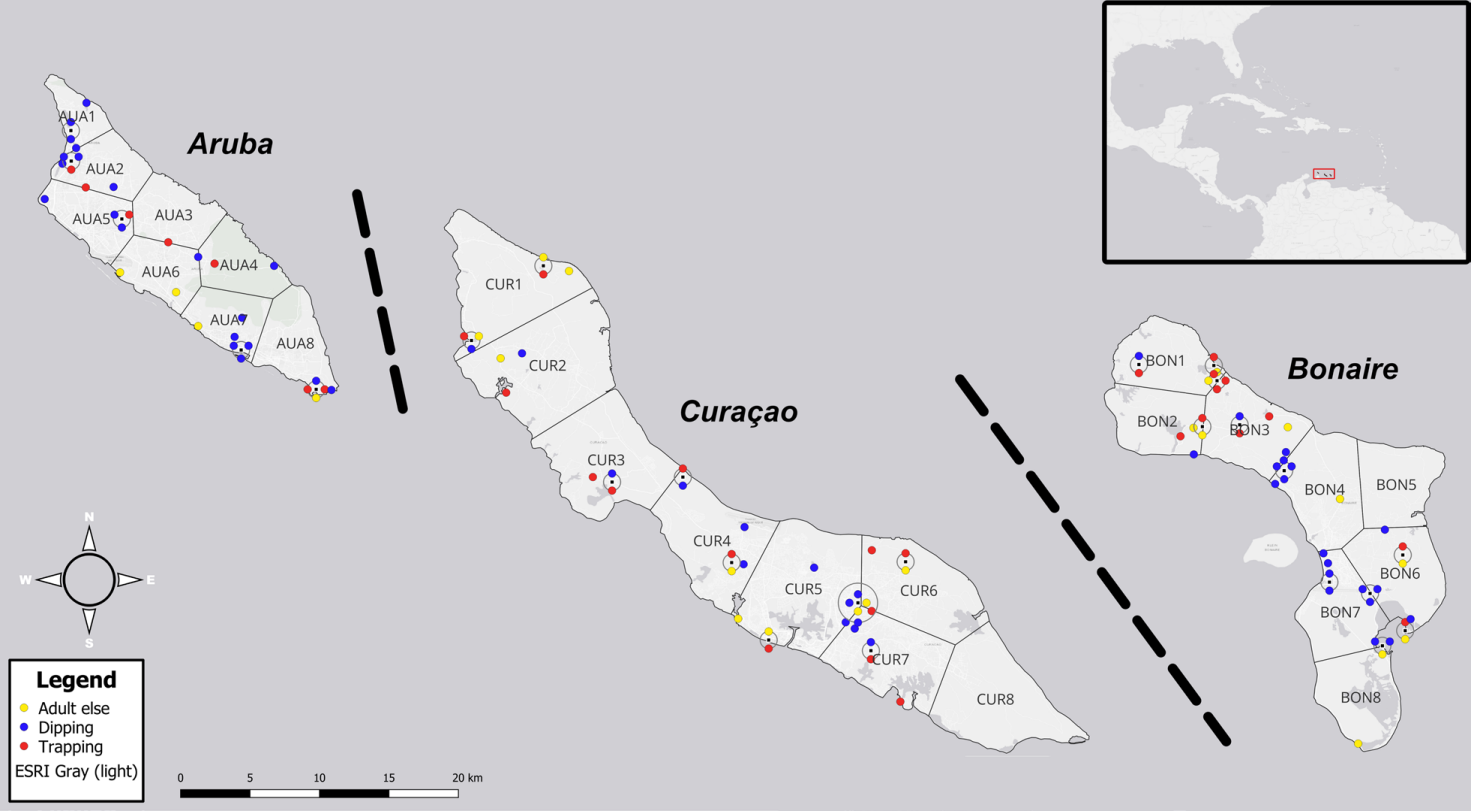
Sample locations and island sections used in this study. Coloured dots represent sample locations for all mosquito specimens from which sequences were obtained for the six studied species. Colour of each dot represents the applied sampling method (see Additional File 1: Supplementary Table 1 for sample sizes per sampling method). ‘Adult else’ encompasses adult mosquitoes collected by human landing catches or by netting. ‘Dipping’ includes both sampled larvae and pupae (the latter were sampled for DNA lab work as adults). Sample locations in close proximity are presented in concentric circles. Depicted sections are the equally sized island sections which were created for mosquito subsampling in this study. Dashed lines indicate that the distance between islands is not to scale. Inset in top right corner shows location of the islands within the Caribbean, ~30–80 km off the coast of Venezuela. Basemap source: ESRI (Environmental Systems Research Institute Inc., Redlands, California)

The variation in genetic diversity and population genetic structure among ecologically diverse mosquito species was explored on the islands of Aruba, Curaçao and Bonaire in November and early December 2022 (Fig. 1), during the ‘Expedition ABC Mug-Sangura 2022’. Aruba (180 km^2^) and Bonaire (288 km^2^) were sampled for 6 days, while Curaçao, a slightly larger island (444 km^2^), was sampled for 8 days. The mosquito samples used in the genetic analysis were obtained from the larger collections made during this expedition. Some additional samples from Bonaire were obtained from the ‘Naturalis Relay Expedition 2022–2023’, collected between 1 and 14 December 2022. The islands are located in the southern Caribbean Sea, ~ 30–80 km off the coast of Venezuela, and follow a west to east gradient, with Aruba and Curaçao ~80 km apart and Curaçao and Bonaire ~45 km apart (Fig. 1). All three islands have a semi-arid tropical savannah climate and offer a rich diversity of habitats, including dry tropical forests, streams, freshwater and saltwater lakes, mangroves, caves, rocky and sandy shores, as well as various urban habitats [36]. The fieldwork was carried out during the late rainy season, which typically lasts from October to December/January on the islands [38], with heavy rainfalls during the months preceding the fieldwork [39, 40], likely causing high densities of mosquitoes.

### Sampling strategy

The mosquitoes included in this study were sampled using a variety of trapping methods (Additional File 1: Supplementary Table 1) to collect individuals from different life stages and habitats, thus increasing the chance of a thorough sample of the genetic variation within local populations. Adult mosquitoes were trapped using CO_2_-baited BG Pro traps (Biogents, Regensburg, Germany), which were set up in 31 locations (Fig. 1) during daytime and emptied the next morning. The traps were used as Encephalitis Virus Surveillance (EVS) style traps by hanging them approximately 100 cm [41] above the ground in a sheltered place (e.g., within vegetation). CO_2_ was generated through sugar fermentation, utilising a mixture of beet sugar, active-dry yeast, yeast nutrient salt and tap water. Additionally, 24 locations (Fig. 1) were sampled for adult mosquitoes through human landing catches or by catching flying and resting mosquitoes with a net and aspirator. Larval sampling was performed by primarily using 350 ml Mosquito Dippers (BioQuip Products, Rancho Dominguez, California); smaller water bodies were sampled with turkey basters, soup spoons, or manual hand suction pumps [42]. Larval samples (both larvae and pupae) were taken from a diverse range of water bodies (*n* = 53), such as ponds, lakes, seashore water bodies, streams, rainwater puddles, rock pools, crab holes, bromeliads, tree holes and artificial containers. Mosquitoes were collected from different areas on the islands, both coastal and inland regions, aiming for a wide coverage of the entire islands (Fig. 1). Suitable terrestrial and aquatic sampling sites were identified on sight or selected based on arial maps and expertise of local collaborators.

Upon collection, live specimens were put in the freezer for at least 20 min prior to morphological identification. Collected pupae were kept in falcon tubes with sampled water until they emerged as adults before putting them in the freezer. Identification was done morphologically with an unpublished identification key for the islands, which was constructed based on existing literature and keys for the region ([37, 43–48]). After identification, specimens were stored in 70% ethanol, and several undamaged adult specimens were mounted.

### Taxon selection and specimen selection

To obtain reliable insights into the local variation in mosquito population structure and genetic diversity on the islands, species were selected according to the following three criteria: (1) they were among the most abundant species on the islands, (2) occupied different ecological niches and (3) constituted a mix of native and non-native mosquito species. Of the 16 species of mosquitoes recorded during the expedition (Additional file 1: Supplementary Table 2), nine species were observed only on a single island or in a limited number of locations and were consequently excluded from further analysis. Based on the criteria, specimens of six species were included in this study, which represent a variety of ecological strategies: *Ae. aegypti*, *Ae. taeniorhynchus*, *Cx. nigripalpus* Theobald, 1901, *Cx. quinquefasciatus*, a currently undescribed species of *Deinocerites*, and *Haemagogus chrysochlorus* Arnell, 1973 (see Additional File 1: Supplementary Table 3). The native species include two species breeding in dynamic and temporary water bodies (*Cx. nigripalpus*, breeding in temporary freshwater bodies, and *Ae. taeniorhynchus*, breeding in coastal temporary water bodies) and two species with highly specialised breeding habitats (*Deinocerites* sp., breeding in crab burrows in the mangrove, and *Hg. chrysochlorus*, breeding in tree holes) [37, 45, 49]. The non-native species *Ae. aegypti* and *Cx. quinquefasciatus* are opportunistic container breeders with a strong association to urban areas [37, 45]. Both are considered to have invaded and colonised the Caribbean in the sixteenth century [50, 51].

The aim was to include between 30 and 60 specimens per species in total for genetic analysis. To ensure a geographical spread of data points on each island, specimens were randomly subsampled from eight sections per island. A polygon for each island was manually created, before being divided into eight equally large sections by performing *K*-means clustering in QGIS (version 3.28.2 Firenze) on a random point layer (100,000 points) within the island polygon. By creating Voronoi polygons and taking the intersect with the island polygon, new polygons for eight equally large sections per island were created (Fig. 1). For each island section, a sample location was randomly selected using the ‘sample’ function from R Base in RStudio (version 2022.12.0 Build 353; R version 4.2.1). From every selected location, three specimens were taken, preferably adult samples as adult DNA extractions had significantly higher success rates than larval extractions during a DNA extraction pilot in the lab. If fewer than three specimens of a given species were collected at a selected location, additional specimens were randomly subsampled to obtain three specimens per species per island section, aiming for an even distribution of the number of specimens per island section. Ultimately, the total number of specimens differed per species or per island, because of locally low abundance of some species on specific islands or technical difficulties during the extraction or sequencing phase (Table 1).

**Table 1:** Total numbers of specimens per island and per species included in the present study

Species	*n*_*Aruba*_ (*n*_*locations*_)	*n*_*Curaçao*_ (*n*_*locations*_)	*n*_*Bonaire*_ (*n*_*locations*_)	*n*_*total*_ (*n*_*locations*_)
*Aedes aegypti*	6 (*4*)	7 (*4*)	7 (*4*)	20 (*12*)
*Aedes taeniorhynchus*	12 (*7*)	13 (*7*)	13 (*7*)	38 (*21*)
*Culex nigripalpus*	21 (*8*)	18 (*7*)	19 (*6*)	58 (*21*)
*Culex quinquefasciatus*	21 (*12*)	17 (*9*)	14 (*8*)	52 (*29*)
*Deinocerites* sp.	23 (*7*)	16 (*4*)	15 (*4*)	54 (*15*)
*Haemagogus chrysochlorus*	2 (*1*)	13 (*8*)	21 (*13*)	36 (*22*)
Total	85 (*39*)	84 (*39*)	89 (*42*)	258 (*109*)

Note that the total number of locations is not equal to the sum of either the number of locations per species or the number of locations per island, since a number of specimens from different species were sampled at the same location, for example, in a BG Pro Trap

The specimens are all vouchered and stored in the Culicidae collection of Naturalis Biodiversity Center, formerly the National Museum of Natural History, Leiden, the Netherlands (RNMH).

### DNA extraction and amplification

To elucidate patterns in the population genetics of local mosquito populations, mitochondrial DNA (mtDNA) was used. Since the haploid mitogenome is exclusively inherited maternally [52], the effective population size is four times smaller than in nuclear DNA, resulting in faster lineage sorting [53–55]. Theoretically, this enables mtDNA to reflect changes in population structure on shorter time scales [56], increasing chances of detecting changes in population structure. Based on an unpublished dataset of 83 mitochondrial genomes of 27 Dutch mosquito species, four genes—*COI*, *COII*, *ND4* and *ND5* e.g., [32, 57–59]—which were previously used in other mosquito population genetic studies involving mitochondrial markers, were tested for their nucleotide substitution rates (Additional File 1: Supplementary Fig. 1). Of these four genes, we selected the *COII* gene as marker as it showed the highest synonymous nucleotide substitution rate (K_s_). Synonymous mutations are neutrally inherited because they do not alter the protein sequence or function, making them largely unaffected by natural selection. As a result, they are more likely to reflect demographic history (e.g., invasiveness, migration and population bottlenecks) and population structure on islands, as they capture stochastic signatures such as genetic drift and the accumulation of neutral variation over time.

Extractions on adult specimens were carried out using a single leg, which was rinsed with ddH_2_O for 10 min and dried (adapted from [60]). DNA extraction was performed using 20 μl Lucigen QuickExtract DNA Extraction Solution (Lucigen, Middleton, Wisconsin), following the manufacturer’s protocol with the following adaptations: the first incubation at 65 °C for 15 min, and the second incubation at 98 °C for 2 min. For larval specimens, one to two segments cut from the abdomen were used or the entire abdomen for tiny larvae. Larval DNA extraction had low success rates using Lucigen QuickExtract and was therefore performed using the Higher Purity Tissue DNA purification kit (Canvax Biotech, Valladolid, Spain), following the protocol of the manufacturer. DNA of *Cx. quinquefasciatus* larvae was extracted using a DNeasy Blood and Tissue kit (QIAGEN, Hilden, Germany) following the manufacturer’s protocol, with the adaptation of using 50 μl of provided Buffer AE for DNA elution. The Canvax and QIAGEN kits had similar DNA yields. Samples were stored at −20 °C.

Each PCR was prepared with 2.0 μl of DNA extract, 17.5 μl of Hot Start Taq 2× Master Mix (New England Biolabs, Ipswich, Massachusetts), 1.4 μl (10 μM stock) of forward and reverse primer and 12.7 μl of nuclease-free water, adding up to a volume of 35 μl per reaction. The three primer sets used varied for different species (Table 2), but all targeted the same 745-bp locus. One newly developed reverse primer was utilised during this study (Cul-COII-R) (Table 2). The forward primer annealing site was located in tRNA-Leu DNA, and the reverse primer annealing site was in tRNA-Lys, resulting in an amplicon that included the *COII* gene. The PCR protocol was the same for all three primer sets, except for the annealing temperature (*T*_a_) (see Table 2): 30 s of initial denaturation at 95 °C, followed by 35 cycles of 30 s denaturation at 95 °C, 30 s annealing and 1 min extension at 68 °C, concluded with 5 min final extension at 68 °C. All PCR products were checked on 1% agarose gels, before sending out for Sanger sequencing. DNA fragments were sequenced in both directions.

**Table 2:** List of primer sets used per species with primer details

Species	Primer set (reference)	Primer sequence (5′–3′)	*T*_a_ (°C)
*Aedes aegypti*	F: SCTL2-J-3037 [87]	ATGGCAGATTAGTGCAATGA	47
	R: Ae-COII-R [32]	GATTTAAGAGATCATTACTTGC	
*Aedes taeniorhynchus*	F: SCTL2-J-3037	ATGGCAGATTAGTGCAATGA	47
	R: Ae-COII-R	GATTTAAGAGATCATTACTTGC	
*Culex nigripalpus*	F: SCTL2-J-3037	ATGGCAGATTAGTGCAATGA	47
	R: Cul-COII-R (†)	GRTTTAAGAGAYCAKTACTTGC	
*Culex quinquefasciatus*	F: SCTL2-J-3037	ATGGCAGATTAGTGCAATGA	45
	R: TK-N-3785 [87]	GTTTAAGAGACCAGTACTTG	
*Deinocerites* sp.	F: SCTL2-J-3037	ATGGCAGATTAGTGCAATGA	47
	R: Cul-COII-R (†)	GRTTTAAGAGAYCAKTACTTGC	
*Haemagogus chrysochlorus*	F: SCTL2-J-3037	ATGGCAGATTAGTGCAATGA	47
	R: Cul-COII-R (†)	GRTTTAAGAGAYCAKTACTTGC	

For each primer set, the forward (F) and reverse (R) primers are given. ^†^Newly developed reverse primer for this study

The Sanger-sequencing data of *Ae. taeniorhynchus* specimens contained multiple conflicting base calls in specific positions in the forward and reverse sequences, potentially resulting from nuclear mitochondrial DNA segments (NUMTs) [61, 62]. To eliminate ambiguities, all samples of this species were sequenced again using Oxford Nanopore sequencing [63]. Nanopore sequences a single DNA fragment, allowing us to analyse the individual reads at locations that otherwise returned double peaks in the chromatogram derived from Sanger sequencing. During the first PCR, the ONT-CO2F/ONT-Ae-COII-R primers were used (forward primer: 5′-TTTCTGTTGGTGCTGATATTGCATGGCAGATTAGTGCAATGA-3′ and reverse primer: 5′-ACTTGCCTGTCGCTCTATCTTCGATTTAAGAGATCATTACTTGC-3′), followed by a second PCR using the Oxford EXP-PBC096 Barcode kit and LongAmp Taq 2× master mix. Every PCR was followed by sample quality checks on E-Gel and tapestation and a bead clean-up with MN-beads. After end repair, another MN-bead clean-up and ligation of the Sequencing Adapters with the Oxford SQK-LSK114 Ligation Sequencing Kit v14, the DNA was loaded onto a Flongle flow cell for sequencing using Oxford Nanopore GridION.

### Sequence alignment and analysis

Sequence quality control, trimming and alignment were performed using Unipro Ugene software (version 45.1; [64]). For the Sanger sequencing read files, the forward and reverse reads were aligned, and primers as well as low-quality ends were trimmed. Sequences with high levels of ambiguity were excluded from the analysis. The raw Nanopore reads of *Ae. taeniorhynchus* were aligned to a reference, and a consensus sequence was generated using the most common nucleotide at each position. All sequences were trimmed to remove the tRNA flanks and to obtain full sequences of the *COII* marker gene (684 bp). Sequences with many ambiguities were excluded from the analysis. Finalised sequences were aligned using the MUSCLE default algorithm [65] and converted to Nexus-format text-files as described by Leigh et al. [66]. All finalised sequences are available on BOLD [67] (‘Availability of Data and Materials’ section).

Haplotype network analysis was performed using PopART software (version 1.7; [68]) to infer genealogical relationships among the studied specimens. Haplotype networks were plotted per species using the median-joining network (MJN) inference method [69]. The number of haplotypes (*H*) and the number of segregating sites (*S*) were also retrieved from PopART. Additionally, all species were plotted in a single haplotype network (Additional file 1: Supplementary Fig. 2) using the TCS method [70], which reduced the formation of complex knots in between species in the network.

In addition to haplotype network analysis, DnaSP software (version 6.12; [71]) was used to calculate the nucleotide diversity (*π*), haplotype diversity (*Hd*), Tajima’s *D* and Fu’s *F*_*S*_ per species. Tajima’s *D* estimates if mutations occur due to neutral evolution or selective pressures by taking the difference between observed and expected nucleotide diversity [72]. Fu’s *F*_*S*_ statistic is a neutrality test similar to Tajima’s *D* but uses the haplotype distribution [73]. Since these statistics are both influenced by changes in population size, it can be used to detect past population expansions. Negative values for both neutrality tests represent an excess frequency of rare polymorphisms or alleles, indicative of recent population expansion or genetic hitchhiking [74]. Two positions were masked for *Ae. aegypti* sequences in PopART and DnaSP, due to ambiguous base calls. To check if the included specimens were correctly identified, a phylogenetic tree including all successful sequences was reconstructed. IQ-Tree2 [75] was used to calculate a maximum likelihood tree, using standard model selection with ModelFinder [76] and 1000 Ultrafast bootstraps [77]. The TPM2u + F + G4 was selected as best-fit model according to the BIC value by ModelFinder. Intraspecific branching is depicted as a triangular radiation (henceforth referred to as ‘collapsed’ branching). A wider triangle represents longer internal branch lengths in the respective collapsed clade. Branch length represents genetic distance between specimens. Support values are given per node as bootstrap values (%). The calculated tree was visualised using packages ‘ggtree’ (version 3.10.0; [78]) and ‘phytools’ (version 2.0–3; [79]) in R (version 2023.12.0 Build 369; R version 4.3.2).

## Results

### Mosquito collection

In total, *COII* sequences of 258 mosquitoes belonging to six species were successfully obtained during this study. Sequences were obtained from mosquitoes collected from a total of 109 different sampling locations (all species combined; Table 1), with a wide geographical coverage of each island, and each species was collected on all three islands (Fig. 1 and Additional file 1: Supplementary Figs. 3 and 4). Roughly equal numbers of specimens were used from each island for all species except *Hg. chrysochlorus*, which was found only in a single location in Aruba. All species were resolved as monophyletic in the reconstructed phylogenetic tree (Additional File 1: Supplementary Fig. 5), confirming that the species are indeed genetically separated populations. The distances between the haplotype networks are given in Additional File 1: Supplementary Fig. 2.

### Genetic diversity and population genetic structure

**Fig. 2:**
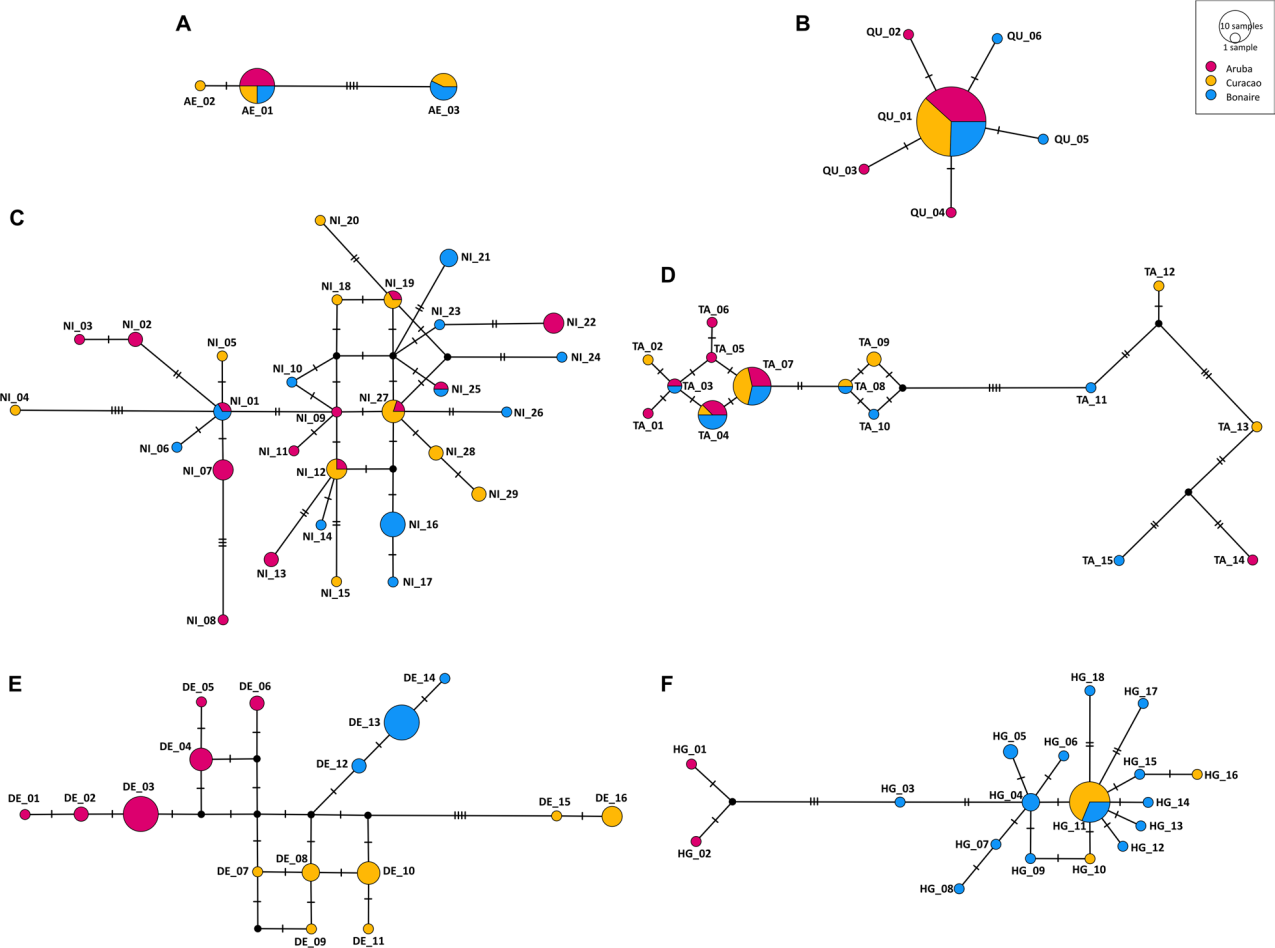
Haplotype network analysis of *COII* sequences using MJN inference for (**A**) *Aedes aegypti*, (**B**) *Culex quinquefasciatus*, (**C**) *Culex nigripalpus*, (**D**) *Aedes taeniorhynchus*, (**E**) *Deinocerites* sp. and (**F**) *Haemagogus chrysochlorus*. Pie charts represent unique haplotypes found in this study, with pie chart size representing the number of sequences with the same haplotype and pie chart colours corresponding to the island of origin of the sequences (pink: Aruba; yellow: Curaçao; blue: Bonaire). The hatch marks on the edges represent the number of genetic differences between closely related sequences

The analyses revealed three main patterns of genetic diversity and differentiation among the six species, varying in the degree of intraspecific genetic diversity and the species-specific population genetic structure. The first group of species, consisting of *Ae. aegypti* and *Cx. quinquefasciatus*, has a low genetic diversity, which largely overlaps between the three islands (Fig. 2A and B). For these species, the haplotype network analysis revealed a total of three and six unique haplotypes, respectively. Low estimates for the nucleotide diversity (*π*) (0.00296 and 0.00028, respectively) and haplotype diversity (*Hd*) (0.542 and 0.185, respectively) were calculated compared with the other studied species (Table 3). Regarding the neutrality tests, Tajima’s *D* was not significant in *Ae. aegypti* but negative and significant (*P* < 0.05) in *Cx. quinquefasciatus*. Fu’s *F*_*S*_ was positive in *Ae. aegypti* and negative in *Cx. quinquefasciatus*, in congruence with Tajima’s *D* estimate for this species (see ‘Methods’ section for an explanation). *Aedes aegypti* consists of two dominant haplotypes (AE_01 & AE_03), which together comprise all specimens except for one (AE_02). For *Cx. quinquefasciatus*, one dominant haplotype (QU_01) was found, shared by 47 specimens. Five more haplotypes were found, but these were present only in a single mosquito, differing by only one single substitution from the dominant haplotype. As a result, the haplotype network shows a subtle star-shaped structure.

**Table 3:** Summary of the population genetic statistics of the mitochondrial *COII* sequences for all six studied species

Species	*COII*	*H*	*S*	*π*	*Hd*	*s* (*Hd*)	*D*	*P (D)*	*F*_*S*_
*Aedes aegypti*	20	3	5	0.00296	0.542	0.076	1.31665	NS	3.244
*Culex quinquefasciatus*	52	6	5	0.00028	0.185	0.072	−1.98592	*	−6.970
*Culex nigripalpus*	58	29	28	0.00644	0.966	0.009	−1.04344	NS	−16.792
*Aedes taeniorhynchus*	38	15	22	0.00612	0.826	0.050	−0.67506	NS	−3.188
*Deinocerites* sp.	54	16	18	0.00606	0.885	0.024	0.15591	NS	−2.636
*Haemagogus chrysochlorus*	36	18	21	0.00320	0.803	0.068	−1.92392	*	−13.105

For each species: the number of sequences included in the calculation (*COII*), the number of haplotypes (*H*), the number of segregating or polymorphic sites (*S*), the nucleotide diversity (*π*), the haplotype diversity (*Hd*), the standard error of the haplotype diversity (*s* (*Hd*)), estimated Tajima’s *D* (*D*), the *p*-value for the estimated Tajima’s *D* (*P (D)*) and estimated Fu’s *F*_*S*_ (*F*_*S*_). Significance shown as *P < 0.5; NS, not significant

The second species group, consisting of *Cx. nigripalpus* and *Ae. taeniorhynchus*, has a high genetic diversity. However, haplotypes did not cluster per island but rather showed many connections between haplotypes from different islands. For these species, a total of 15 and 29 unique haplotypes, respectively, were resolved (Fig. 2C and D). This was supported by the high estimates for the nucleotide diversity (0.00612 and 0.00644, respectively) and haplotype diversity (0.826 and 0.966, respectively), which are much higher than for the first species group (Table 3). Regarding the neutrality tests for these species, Tajima’s *D* was not significant, and Fu’s *F*_*S*_ was negative (see ‘Methods’ section for an explanation). For *Ae. taeniorhynchus*, the haplotype network shows that the majority of the specimens is clustered on the left side of the network (TA_01 to TA_10), but considerable genetic distances are present within the network, even between specimens from the same island. For *Cx. nigripalpus*, a more complex, partly reticulated network was recovered with five haplotypes found on multiple islands (NI_01, NI_12, NI_19, NI_25, and NI_27).

The third species group, consisting of *Deinocerites* sp. and *Hg. chrysochlorus*, is characterised by a high genetic diversity. In contrast to the second species group, most haplotypes were found only on a single island. For these species, 16 and 18 unique haplotypes, respectively, were resolved (Fig. 2E and F). Additionally, haplotype diversity estimates were high for both species (0.885 and 0.803, resp.). The nucleotide diversity, however, had a high estimate in *Deinocerites* sp. (0.00606), but a lower estimate for *Hg. chrysochlorus* (0.00320) (Table 3). Similarly, Tajima’s *D* was not significant for *Deinocerites* sp. but negative and significant for *Hg. chrysochlorus* (*P* < 0.05), and Fu’s *F*_*S*_ was negative for both species (see ‘Methods’ section for an explanation). All haplotypes found for *Deinocerites* sp. were island specific and clustered together per island into four groups in the haplotype network: one group with all haplotypes from Aruba (DE_01 to DE_06), two groups with only haplotypes from Curaçao (DE_07 to DE_11 and DE_15 to DE_16) and one group with all haplotypes from Bonaire (DE_12 to DE_14). For *Hg. chrysochlorus*, all haplotypes were island specific as well, except for the relatively dominant haplotype (HG_11), which was found on Curaçao and Bonaire. Although population structure in the network was not as distinct as for *Deinocerites* sp., it shows a distinct cluster of haplotypes from Aruba and a star-shaped structure centred around the dominant haplotype.

## Discussion

The central aim of this study is to explore the potential generalities in variation in mosquito population genetics among a comprehensive assemblage of native and non-native mosquito species. To this end, we investigated the population genetics of an ecologically diverse set of mosquito species from three different Caribbean islands, including both native and non-native species, using the mitochondrial *COII* marker. The haplotype network analysis revealed three groups of species, which differed profoundly in their degree of genetic diversity and the geographical clustering of similar haplotypes. In line with our hypothesis, the populations of *Ae. aegypti* and *Cx. quinquefasciatus*, both non-native species, exhibit lower genetic diversity compared with native mosquitoes. Among the native species, we found that species with ecologically well-defined breeding habitats that are not widely available (e.g., highly specialised natural container breeding habitats for *Deinocerites* sp. and *Hg. chrysochlorus*) show a high degree of geographical clustering of similar haplotypes compared with species with more widely available habitats. This suggests that the population genetics of mosquitoes primarily vary along a historical axis, while also varying along an ecological axis among native species. Overall, these results highlight that mosquito population genetics can differ strongly between native and non-native populations, even within a confined area such as the Dutch Leeward Antilles that has a relatively low species diversity allowing us to only analyse a small set of species that are in common.

Among the studied species, both *Ae. aegypti* and *Cx. quinquefasciatus* stood out due to their low genetic diversity compared with the four other species based on the mitochondrial *COII* marker. Only three and six haplotypes were found for these two non-native species, respectively, while the native species had almost 20 unique haplotypes on average (Table 3). These results from the Americas, together with low estimates for both the nucleotide diversity and the haplotype diversity (especially for *Cx. quinquefasciatus*)*,* differ from the much higher levels of genetic diversity found in originally native populations for both species (e.g., *Ae. aegypti* in Africa [80] and *Cx. quinquefasciatus* in India [24, 81]). Since both *Ae. aegypti* and *Cx. quinquefasciatus* have likely reached the Caribbean in the early sixteenth century, along with the slave trades to the Americas [50, 51], their local populations have had a much shorter period to accumulate mutations. For both species, the low haplotype diversity also suggests a (very) low initial propagule pressure and/or strong selection for specific haplotypes that managed to survive the long journey to the islands. Furthermore, the haplotype network of *Cx. quinquefasciatus* (Fig. 2B) shows a structure consisting of one highly dominant haplotype supplemented by five alternative haplotypes, differing by only a single point mutation from the dominant haplotype. Such a star-shaped structure, together with the significant negative Tajima’s *D* estimate and negative Fu’s *F*_*S*_ estimate (Table 3), may indicate a past founder effect for this species. The scarcity of genetic diversity might also be attributed by a past selective sweep, explaining the absence of a star-shaped cluster in the haplotype network of *Ae. aegypti* (although low sample size cannot be ruled out as potential explanation for the pattern observed here). However, given that selection is much more likely to affect genetically more diverse populations [82], a founder effect remains more probable if *Ae. aegypti* populations have never had high genetic diversity on these islands due to their recent colonisation of probably few individuals. These indications of a past founder effect, together with low levels of genetic diversity in relatively young populations, suggest that population history has had an important role in population genetics of these mosquito species.

Among the four presumed native species with high genetic diversity on the mitochondrial *COII* marker, the inferred haplotype networks show a marked contrast, supporting a subdivision into two species groups that differ in their ecologies. The haplotype networks of both *Cx. nigripalpus* and *Ae. taeniorhynchus* reveal complex reticulation of closely related haplotypes observed on multiple islands. Especially in *Cx. nigripalpus*, many haplotypes that differed only by a single mutational step were found on neighbouring islands, rather than on the same island, and several haplotypes were present on multiple islands. Additionally, three haplotypes were found (NI_01, NI_25 and TA_03 in Fig. 2C-D), which were only collected on Aruba and Bonaire, even though these islands are separated by Curaçao on a west–east gradient. By contrast, the haplotypes of *Deinocerites* sp. and *Hg. chrysochlorus* mostly cluster together per island. This is especially clear for *Deinocerites* sp., as all haplotypes of this species were island specific and haplotypes most closely related grouped together into four sections in the network (Fig. 2E), corresponding to the islands from west to east. For *Hg. chrysochlorus* all haplotypes except the dominant haplotype from Curaçao and Bonaire (Fig. 2F) were island specific, similar to the studied species of *Deinocerites*. This disparity in population genetic structure between these two groups can be contributed to species-specific ecological traits. Both *Cx. nigripalpus* and *Ae. taeniorhynchus* breed in a diverse range of dynamic water bodies [37]. Although the type of temporary water body varies (*Cx. nigripalpus* in permanent and temporary freshwater vegetated pools; *Ae. taeniorhynchus* in coastal marshland, mangroves and beach pools [37, 45, 49]), both species can traverse multiple kilometres to find a bloodmeal and suitable breeding habitat [83, 84], and are thus considered strong flyers [35]. However, *Deinocerites* sp. and *Hg. chrysochlorus* are much more specialised regarding their breeding habitat. These species breed in crab holes and tree holes, respectively [37, 49], which are closely associated with specific and permanent habitats on the islands (mangrove and forest, respectively). Owing to their breeding habitat, the latter two species are (1) more restricted by fixed ranges within the islands and (2) may remain in the same breeding area for several generations. Consequently, such species with clear and narrow niches are less likely to migrate between the islands, leading to a more stratified population genetic structure per island.

This subdivision between both groups of native mosquitoes is in congruence with Becker et al. [85], who distinguished between mosquito species with (1) short dispersal ranges (many container breeders), (2) species that can traverse moderate distances, and (3) those that fly long distances between their breeding habitat and the host’s habitat. Both *Deinocerites* sp. and *Hg. chrysochlorus* fall into the first category, while *Cx. nigripalpus* and *Ae. taeniorhynchus* belong to the third category. Despite *Ae. aegypti* and *Cx. quinquefasciatus* being predominantly container breeders (with *Cx. quinquefasciatus* also found in temporary pools in sparsely vegetated habitat), their short history on the islands hinders direct comparison with the other species, thereby complicating ecological comparisons. This indicates that the population history of non-native species affects the genetic makeup of the population more profoundly than the ecological factors at play, in contrast to locally native species.

The above-mentioned contrasts in mosquito population genetics may act as a proxy for differences in the population dynamics and dispersal patterns among different species. A limited dispersal capacity, especially in combination with a specific breeding habitat, may lead to semi-isolated subpopulations of a mosquito species and decrease the chances of inter-island dispersal, thus promoting higher levels of genetic diversity and a more stratified genetic population structure. This is illustrated by the haplotype network of *Deinocerites* sp. (Fig. 2E), since species in this genus are known to be poor flyers, dispersing not much further than several meters from their crab holes [37]. The haplotype network of *Deinocerites* sp. shows very few connections between haplotypes from different islands and exclusively island-specific haplotypes. This implies that interbreeding of individuals from different islands occurs rarely, suggesting inter-island dispersal to be highly limited. Conversely, species capable of long-distance dispersal hold the potential to sustain at least some degree of gene flow between subpopulations. For both *Cx. nigripalpus*, considered a good flyer (2–4 km), and *Ae. taeniorhynchus*, considered a strong flyer (4+ km) [35], long-distance dispersal may explain the complexity of their haplotype networks. Since three haplotypes were found on both Aruba and Bonaire but not on Curaçao for these species (NI_01, NI_25 and TA_03 in Fig. 2C-D), one might assume that dispersal of these species between the islands was not a natural dispersal event. The presence of closely related haplotypes on different islands may have arisen from a combination of both natural wind-mediated long-distance dispersal and random human-mediated dispersal (i.e., through human means of transport such as airplanes or cars [34]), allowing for local redistribution of mosquitoes of these species within the Dutch Leeward Antilles. Consequently, the sustained high genetic diversity may result from opportunistic breeding habitat selection in newly reached areas, as large parts of the three islands can harbour suitable breeding habitats for these species. Ultimately, this will lead to the formation of subpopulations with gene flow from time to time between the islands.

We acknowledge that using single-locus mitochondrial data provide only a snapshot of genetic diversity. Different genetic markers may show different patterns of genetic diversity, and the maternal inheritance together with a lack of recombination of the mitochondrial genome might result in limited genetic diversity compared with nuclear markers. Even though the combination of these two limitations could potentially conceal certain population genetic patterns, we expect to see similar population genetic patterns as presented here at larger genetic scales, because we observe strong contrasts among the studied mosquito species that link well to ecological theory. The contrasts in population genetics presented here are relevant for many mosquito species that inhabit true islands or island-like systems. The latter includes mainland mosquito populations, as successful dispersal between hosts and suitable breeding habitat in a patchy landscape is fundamental for many mosquito species. Therefore, differences in ecological niche and dispersal capabilities will presumably be reflected in the population genetic structure of mainland mosquitoes similarly to the studied native species on the Dutch Leeward Antilles. This aligns with broader ecological principles, suggesting that similar patterns may emerge in other fragmented or isolated habitats beyond island environments (e.g., [86]).

Above all, owing to the fundamentality of the factors affecting the investigated mosquito population genetics, our results suggest that there might be a contrast between locally non-native and native species worldwide. We suggest that a more comparative approach with multiple species from the same location, without focusing solely on the medically relevant species, helps establish a conceptual framework for understanding how dispersal and habitat fragmentation shape mosquito population genetics in diverse ecosystems.

## Conclusions

Our analyses based on six species from a diverse set of ecological niches show considerable differences in genetic diversity in the mitochondrial *COII* marker between non-native and native species at a specific location, illustrating the role of population history in patterns of mosquito population genetics. Moreover, the results show that, within a pool of native species, major differences in population genetic structure may arise from species-specific ecological characteristics (e.g., breeding habitat specificity and dispersal capacity). Understanding the general drivers of population genetic structure in mosquitoes requires more studies that consider a broader range of species from a single area. This approach will enhance our understanding of the ecological and historical drivers of these patterns and help place the patterns observed in medically relevant target species into a broader context.

## Supplementary Information

Additional file 1: Supplementary Table 1. Overview of the number of specimens for each species by sampling method included in this study. Supplementary Table 2. Preliminary list of mosquito species collected on Aruba, Curaçao and Bonaire, during the 2022 expedition. Supplementary Table 3. Brief overview of ecological characteristics of the species included in this study. Supplementary Fig. 1. Boxplot of pairwise divergence of the K_a_/K_s_ ratio, K_a_, and K_s_ values for four mitochondrial genes based on 89 mitochondrial genomes (27 species) of Dutch mosquito species. Supplementary Fig. 2. Total haplotype network of all 258 *COII* sequences included in this study using TCS inference. Supplementary Fig. 3. All collection localities of specimens included in this study for *Aedes aegypti*, *Aedes taeniorhynchus*, and *Haemagogus chrysochlorus*. Supplementary Fig. 4. All collection localities of specimens included in this study for *Culex quinquefasciatus*, *Culex nigripalpus*, and *Deinocerites* sp. Supplementary Fig. 5. Unrooted Maximum Likelihood tree of the 258 included Caribbean mosquito sequences (684 bp).

## Data Availability

All specimens have been collected in local natural park authorities, and research and collection permits can be presented upon request. Specimens were sampled mostly non-destructively, and the vouchers are stored in the Culicidae collection of Naturalis Biodiversity Center, formerly the National Museum of Natural History, Leiden, the Netherlands (RMNH). All sequences, including trace files, are available in the ‘Caribbean Mosquitoes (CAMOZ)’ project on BOLD (www.boldsystems.org) under accession numbers CAMOZ009-23 – CAMOZ363-24. This study analysed a subset of the data collected during an intensive fieldwork campaign. The complete dataset, which encompasses observations of all other species on the islands, will be published in an taxonomical investigation of the mosquito diversity on the islands.

## Abbreviations

BIC: Bayesian Information Criterion
bp: Basepairs
*COI*: Cytochrome c oxidase subunit I
*COII*: Cytochrome c oxidase subunit II
*D*: Tajima’s *D*
ddH_2_O: Double distilled water
EVS-style trap: Encephalitis Virus Surveillance style trap
*F*_*S*_: Fu’s *F*_*S*_
*H*: Number of haplotypes
*Hd*: Haplotype diversity
K_s_: Synonymous nucleotide substitution rate
MJN: Median-joining network
mtDNA: Mitochondrial DNA
*ND4*: NADH-ubiquinone oxidoreductase chain 4
*ND5*: NADH-ubiquinone oxidoreductase chain 5
NUMTs: Nuclear mitochondrial DNA segments
*π*: Nucleotide diversity
PCR: Polymerase chain reaction
*S*: Number of segregating sites
*T*_a_: Annealing temperature
tRNA-Leu: Transfer RNA for leucine
tRNA-Lys: Transfer RNA for lysine
