# Supplementary material for: The ecological niche and population history shape mosquito population genetics on a group of three Caribbean islands
Source: Parasit Vectors. 2025 May 9;18:167. doi: 10.1186/s13071-025-06801-3 (PMC12065371; doi:10.1186/s13071-025-06801-3)
Supplement: Supplementary file 1 — Additional file 1: Supplementary Table 1. Overview of the number of specimens for each species by sampling method included in this study. Supplementary Table 2. Preliminary list of mosquito species collected on Aruba, Curaçao and Bonaire, during the 2022 expedition. Supplementary Table 3. Brief overview of ecological characteristics of the species included in this study. Supplementary Fig. 1. Boxplot of pairwise divergence of the Ka/Ks ratio, Ka, and Ks values for four mitochondrial genes based on 89 mitochondrial genomes (27 species) of Dutch mosquito species. Supplementary Fig. 2. Total haplotype network of all 258 COII sequences included in this study using TCS inference. Supplementary Fig. 3. All collection localities of specimens included in this study for Aedes aegypti, Aedes taeniorhynchus, and Haemagogus chrysochlorus. Supplementary Fig. 4. All collection localities of specimens included in this study for Culex quinquefasciatus, Culex nigripalpus, and Deinocerites sp. Supplementary Fig. 5. Unrooted Maximum Likelihood tree of the 258 included Caribbean mosquito sequences (684 bp). [file 13071_2025_6801_MOESM1_ESM.docx]

**The ecological niche and population history shape mosquito population genetics on a group of three Caribbean islands**

Pepijn Helleman, Maarten Schrama, Krijn B. Trimbos, Marieta A.H. Braks, Francis Schaffner, Arjan Stroo, Roel M. Wouters, Jordy G. van der Beek*

*Correspondence: jordy.vanderbeek@naturalis.nl

**Supplementary Table 1** Overview of the number of specimens for each species by sampling method included in this study. The overview shows the total number of specimens for each species (*n_total_*), the number of specimens collected in traps (*n_trapped_*), the number of specimens collected by human landing catches (*n_HLC_*), the number of specimens collected as resting mosquitoes using an aspirator (*n_resting_*), and the number of specimens collected by dipping (*n_dipping_*). The number of sampling locations are given for each species by method between parentheses (*n_locations_*).

| Species | *n_total_* (*n_locations_*) | *n_trapped_* (*n_locations_*) | *n_HLC_* (*n_locations_*) | *n_resting_* (*n_locations_*) | *n_dipping_* (*n_locations_*) |
| --- | --- | --- | --- | --- | --- |
| *Aedes aegypti* | 20 (12) | 12 (6) | 1 (1) | 0 (0) | 7 (5) |
| *Aedes taeniorhynchus* | 38 (21) | 3 (2) | 12 (6) | 0 (0) | 23 (13) |
| *Culex nigripalpus* | 58 (21) | 4 (2) | 3 (1) | 0 (0) | 51 (22) |
| *Culex quinquefasciatus* | 52 (29) | 32 (18) | 4 (2) | 4 (2) | 12 (7) |
| *Deinocerites* sp. | 54 (15) | 12 (5) | 6 (3) | 19 (7) | 17 (6) |
| *Haemagogus chrysochlorus* | 36 (22) | 14 (8) | 12 (7) | 0 (0) | 10 (8) |

**Supplementary Table 2** Preliminary list of mosquito species collected on Aruba, Curaçao and Bonaire, during the 2022 expedition. Species of which mitochondrial haplotype data was included in the analyses are given in bold.

| Species | Aruba | Curaçao | Bonaire |
| --- | --- | --- | --- |
| ***Aedes* (*Ochlerotatus*) *taeniorhynchus* (Wiedemann, 1821)** | X | X | X |
| *Aedes* (*Ochlerotatus*) *scapularis* (Rondani, 1848) |  | X | X |
| ***Aedes* (*Stegomyia*) *aegypti* (Linnaeus, 1762)** | X | X | X |
| *Anopheles* (*Nyssorhynchus*) *aquasalis* Curry, 1932 |  | X | X |
| ***Culex* (*Culex*) *nigripalpus* Theobald, 1901** | X | X | X |
| ***Culex* (*Culex*) *quinquefasciatus* Say, 1823** | X | X | X |
| *Culex* (*Culex*) *saltanensis* Dyar, 1928 | X | X | X |
| *Culex* (*Melanoconion*) *conspirator* Dyar & Knab, 1906 |  | X |  |
| *Culex* (*Melanoconion*) *erraticus* (Dyar & Knab, 1906) | X | X | X |
| *Culex* (*Melanoconion*) *unicornis* (Root, 1928) |  | X | X |
| ***Deinocerites* sp. 1** | X | X | X |
| *Deinocerites* sp. 2 | X | X |  |
| ***Haemagogus* (*Haemagogus*) *chrysochlorus* Arnell, 1973** | X | X | X |
| *Psorophora* (*Grabhamia*) *confinnis* s.l. (Lynch Arribálzaga, 1891) | X | X | X |
| *Uranotaenia* (*Uranotaenia*) *lowii* Theobald, 1901 | X | X | X |
| *Wyeomyia* (*Wyeomyia*) *celaenocephala* Dyar & Knab, 1906 |  | X |  |

**Supplementary Table 3** Brief overview of ecological characteristics of the species included in this study.

| Species | Status | Habitat | Disperser; Flight distance |
| --- | --- | --- | --- |
| *Aedes aegypti* | non-native [1] | (artificial) containers [2] | very weak*; average 333 m (0.4 – 2500 m) [3] |
| *Aedes taeniorhynchus* | native | fresh, brackish and saline waterbodies; salt marsh/mangrove [2, 4] | strong; average 31,973 m (10,000 – 48,300 m) [3] |
| *Culex nigripalpus* | native | ephemeral waterbodies, flooded ditches and small ponds [2, 4] | good; average 3267 m (1200 – 4800 m) [3] |
| *Culex quinquefasciatus* | non-native [5] | both (artificial) containers and flooded ephemeral waterbodies [2] | good; average 2184 m (56 - 10,440 m) [3] |
| *Deinocerites* sp. | native | land crab-holes; salt marsh/mangrove [2, 4] | weak; several meters [2]; short flight range only immediate neighbourhood of their breeding sites [6] |
| *Haemagogus chrysochlorus* | native | tree-holes [2] | unknown |

* *Aedes aegypti* natural flight distance is weak, but due to the drought resistant eggs and human mediated dispersal, basically limitless [7, 8].


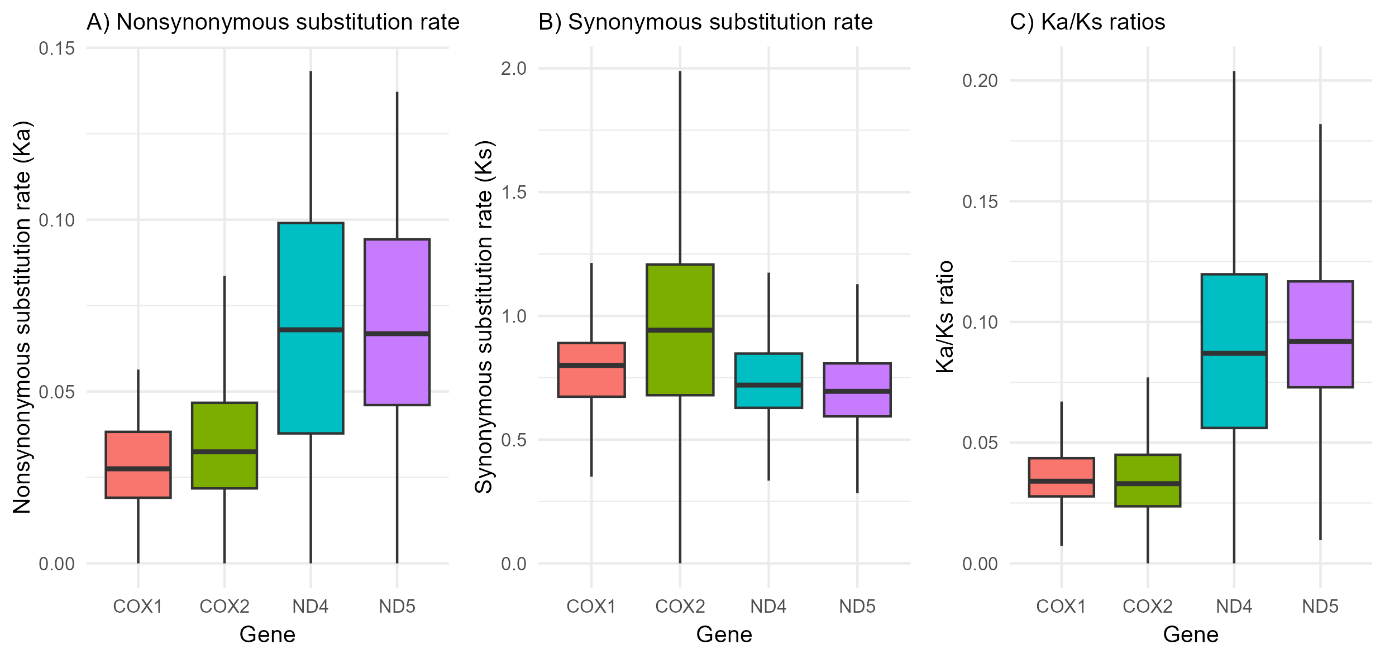


**Supplementary Fig. 1** Boxplot of pairwise divergence of the K_a_/K_s_ ratio, K_a_, and K_s_ values for four mitochondrial genes based on 89 mitochondrial genomes (27 species) of Dutch mosquito species. (**a**) Nonsynonymous substitution rate (K_a_). (**b**) Synonymous substitution rate (K_s_). (**c**) The ratio between the nonsynonymous and synonymous substitution rates (K_a_/K_s_).


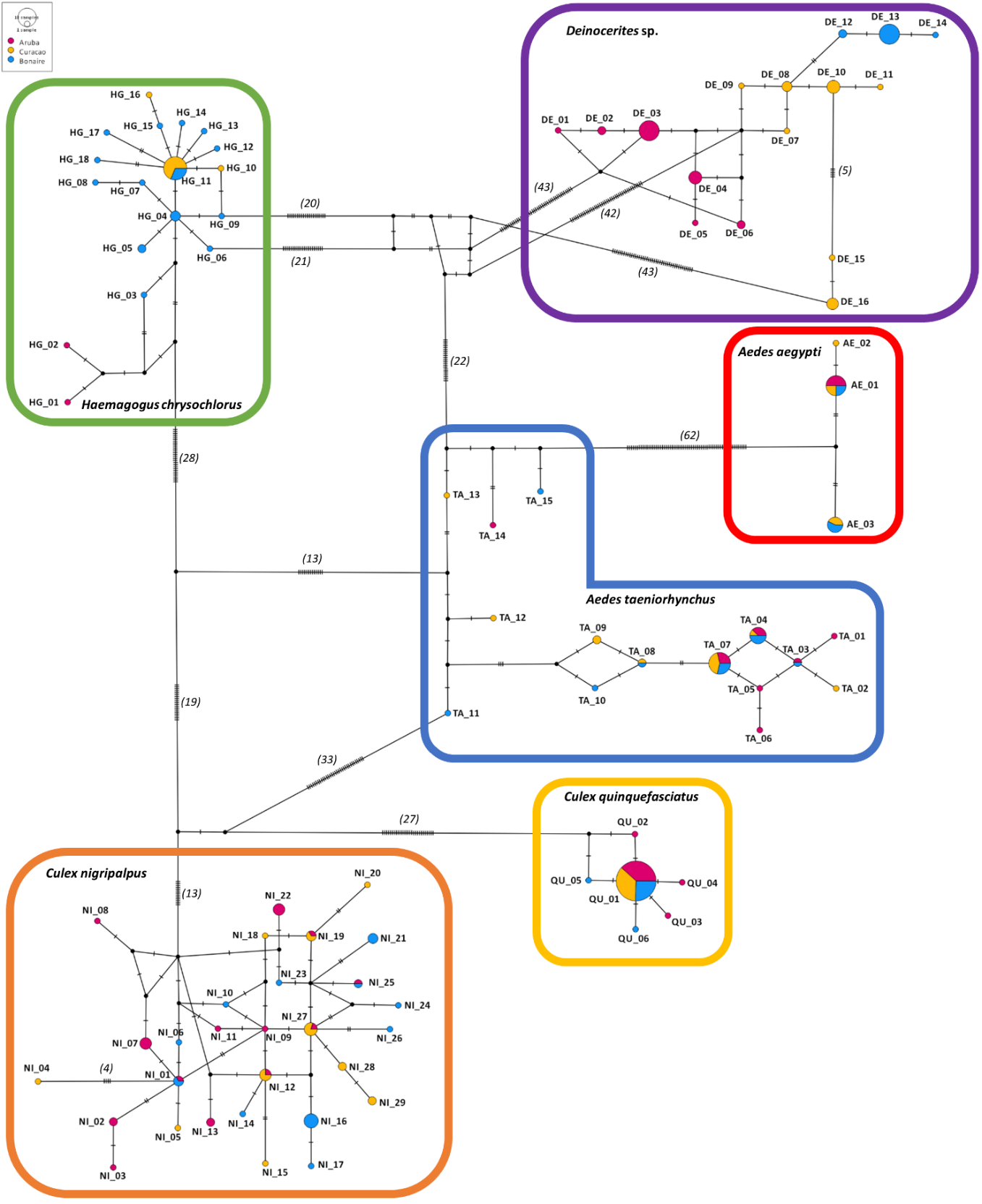


**Supplementary Fig. 2 (previous page)** Total haplotype network of all 258 *COII* sequences included in this study using TCS inference. Species are indicated with coloured frames (green: *Haemagogus chrysochlorus*; purple: *Deinocerites* sp.; blue: *Aedes taeniorhynchus*; red: *Aedes aegypti*; orange: *Culex nigripalpus*; yellow: *Culex quinquefasciatus*). Pie charts represent unique haplotypes found in this study (haplotype name given in bold), with pie chart size representing the number of sequences with the same haplotype and pie chart colours corresponding to the island of origin of the sequences (pink: Aruba; yellow: Curaçao; blue: Bonaire). The hatch marks on the edges represent the number of genetic differences between closely related sequences. Edges representing four or more mutational steps are annotated with the exact number of steps between parentheses.


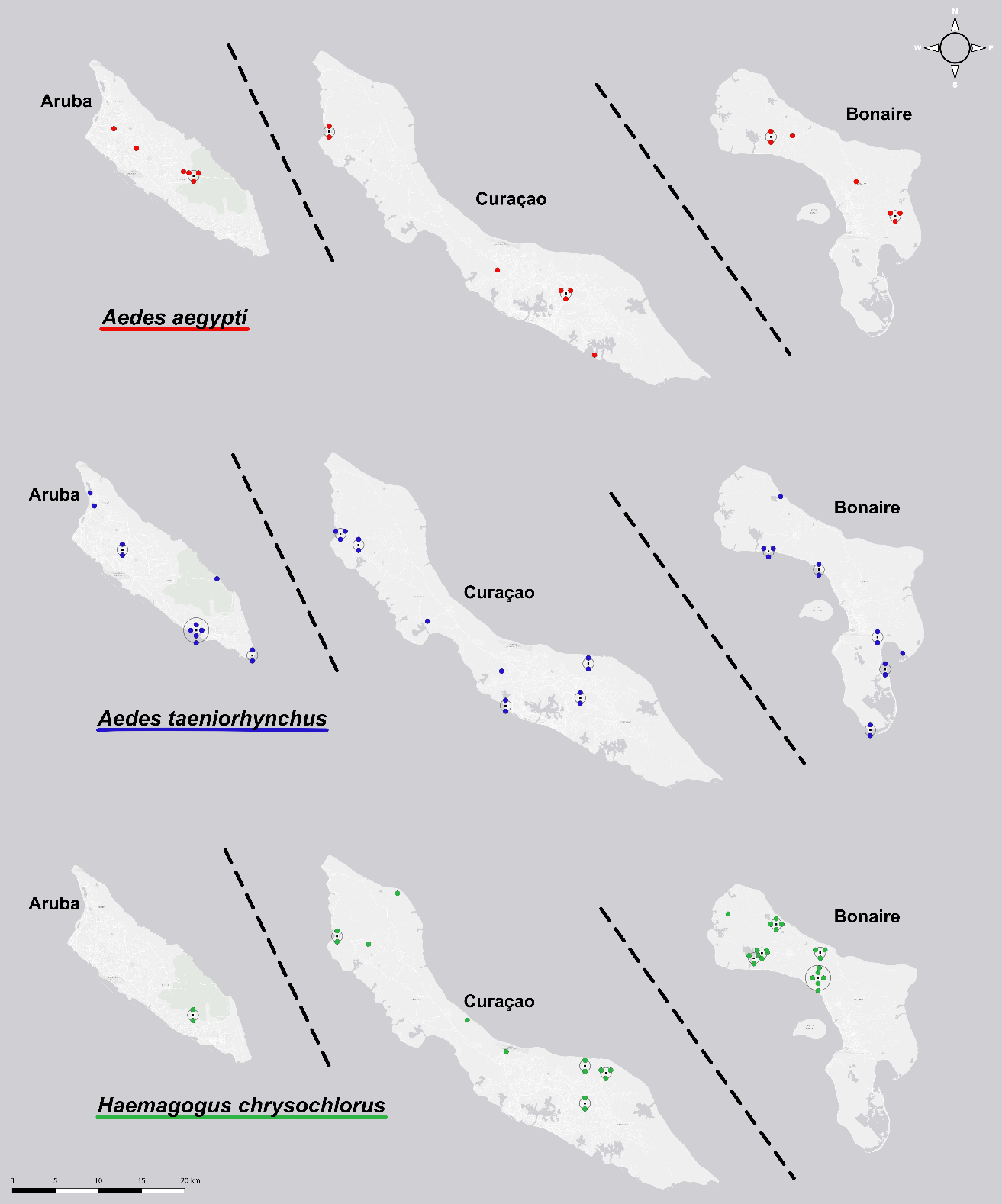


**Supplementary Fig. 3** All collection localities of specimens included in this study for *Aedes aegypti*, *Aedes taeniorhynchus*, and *Haemagogus chrysochlorus* (top to bottom). Colour corresponds with species. Sample locations in close proximity are presented in concentric circles. Dashed lines indicate that the distance between islands is not to scale.


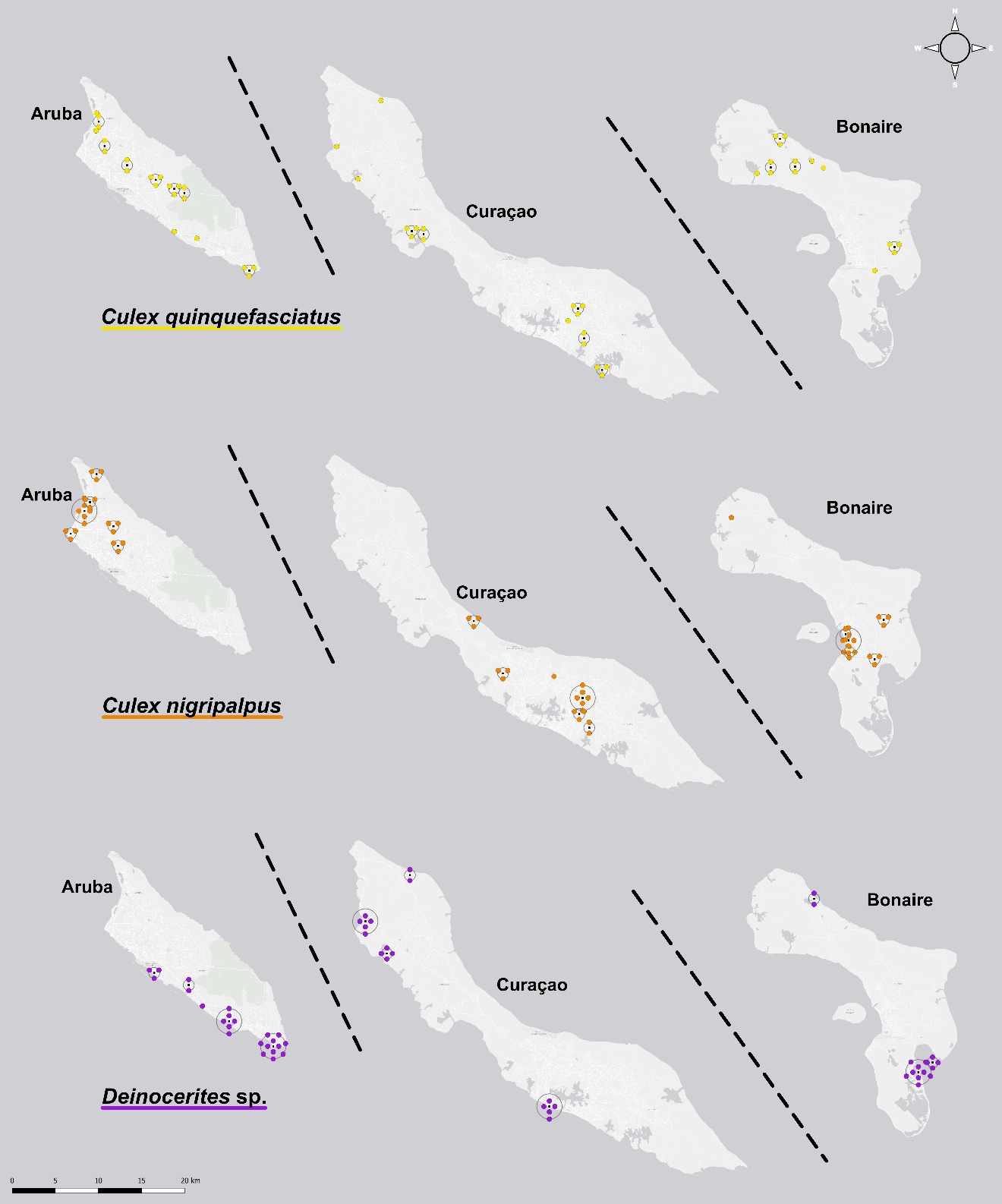


**Supplementary Fig. 4** All collection localities of specimens included in this study for *Culex quinquefasciatus*, *Culex nigripalpus*, and *Deinocerites* sp. (top to bottom). Colour corresponds with species. Sample locations in close proximity are presented in concentric circles. Dashed lines indicate that the distance between islands is not to scale.


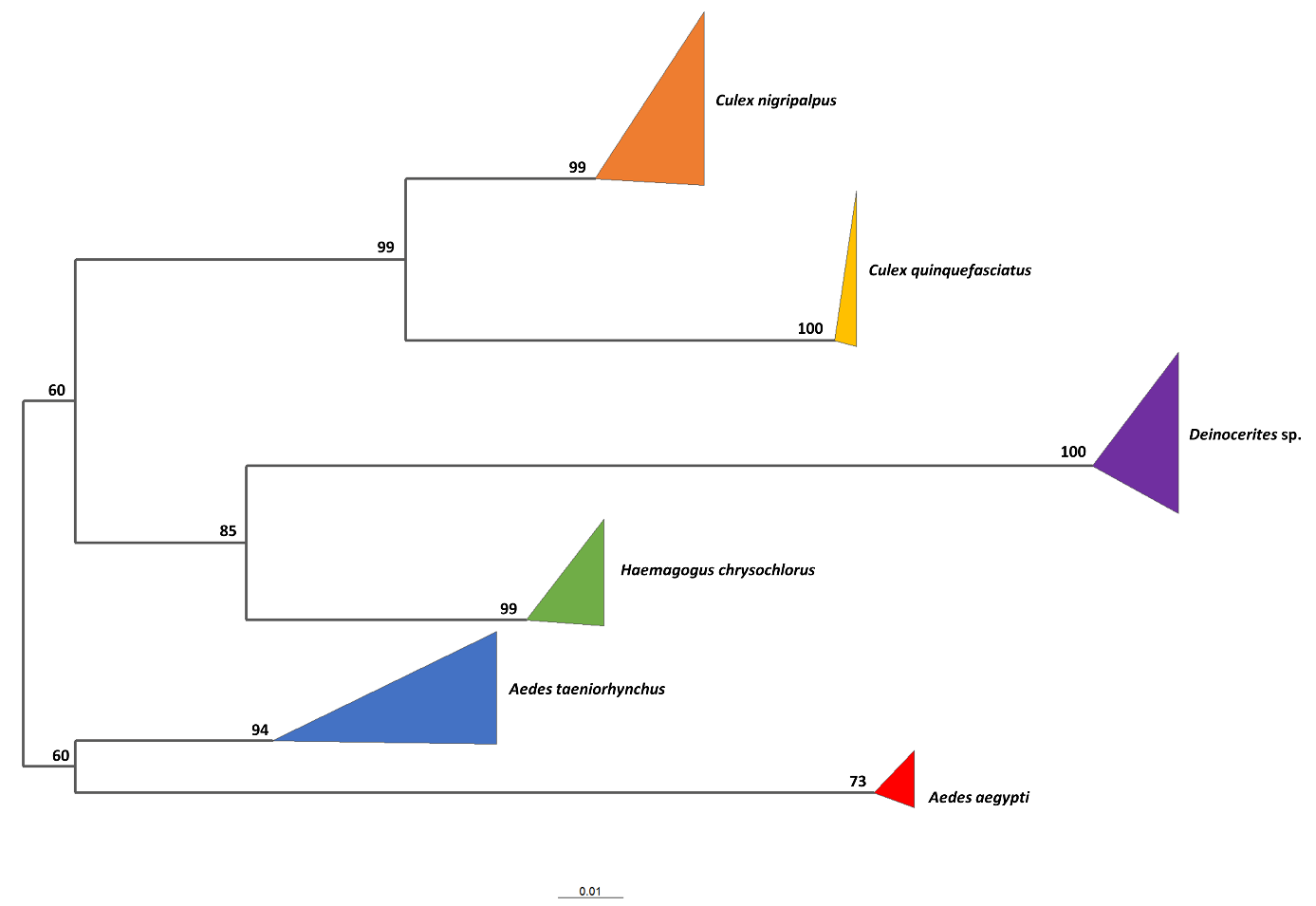


**Supplementary Fig. 5** Unrooted Maximum Likelihood tree of the 258 included Caribbean mosquito sequences (684 bp). Branch length corresponds with the genetic distance between sequences. Branching below the species level is shown as triangular ‘collapsed’ branches. Collapsed branch size corresponds with the internal branch lengths of the clade, and the triangles were scaled to 50% for readability. Support values represent 1000 Ultrafast bootstraps (%).

**References supplementary materials**

1. Powell JR & Tabachnick WJ. History of domestication and spread of *Aedes aegypti*: a review. Mem Inst Oswaldo Cruz. 2013;108(1):11–17. DOI: 10.1590/0074-0276130395
2. Van der Kuyp E. Mosquitoes of the Netherlands Antilles and their hygienic importance. Stud. Fauna Curaçao. 1954;5:37–114.
3. Verdonschot PFM & Besse-Lototskaya AA. Flight distance of mosquitoes (Culicidae): a metadata analysis to support the management of barrier zones around rewetted and newly constructed wetlands. Limnologica. 2014;45:69–79. DOI: 10.1016/j.limno.2013.11.002
4. Wouters RM, Beukema W, Schrama M, Biesmeijer K, Braks MAH, Pepijn Helleman P, et al. Local environmental factors drive distributions of ecologically-contrasting mosquito species (Diptera: Culicidae). Sci Rep 2024;14:19315. DOI: 10.1038/s41598-024-64948-y
5. Belkin JN. The mosquitoes of the South Pacific (Diptera: Culicidae). Vols. 1 and 2. California: University of California Press; 1962.
6. Belkin JN & Hogue CL. A review of the crabhole mosquitoes of the genus Deinocerites (Diptera, Culicidae). Univ Calif publ entomol 1959;14(6):411-458.
7. Fonzi E, Higa Y, Bertuso AG, Futami K & Minakawa N. Human-Mediated Marine Dispersal Influences the Population Structure of Aedes aegypti in the Philippine Archipelago. PLoS Negl Trop Dis 2015;9(6):e0003829. DOI: 10.1371/journal.pntd.0003829.
8. Aedes aegypti - Factsheet for experts. <https://www.ecdc.europa.eu/en/disease-vectors/facts/mosquito-factsheets/aedes-aegypti>. Accessed 20 December 2024.
